# Intradermal Immunization of *Leishmania donovani* Centrin Knock-Out Parasites in Combination with Salivary Protein LJM19 from Sand Fly Vector Induces a Durable Protective Immune Response in Hamsters

**DOI:** 10.1371/journal.pntd.0004322

**Published:** 2016-01-11

**Authors:** Jacqueline Araújo Fiuza, Ranadhir Dey, Dwann Davenport, Maha Abdeladhim, Claudio Meneses, Fabiano Oliveira, Shaden Kamhawi, Jesus G. Valenzuela, Sreenivas Gannavaram, Hira L. Nakhasi

**Affiliations:** 1 Laboratory of Emerging Pathogens, Center for Biologics Evaluation and Research, US Food and Drug Administration, Silver Spring, Maryland, United States of America; 2 Laboratório de Imunologia Celular e Molecular, Centro de Pesquisas René Rachou—Fiocruz Minas, Belo Horizonte, Minas Gerais, Brasil; 3 Vector Molecular Biology Section, Laboratory of Malaria and Vector Research, National Institute of Allergy and Infectious Diseases, National Institutes of Health, Rockville, Maryland, United States of America; New York University, UNITED STATES

## Abstract

**Background:**

Visceral leishmaniasis (VL) is a neglected tropical disease and is fatal if untreated. There is no vaccine available against leishmaniasis. The majority of patients with cutaneous leishmaniasis (CL) or VL develop a long-term protective immunity after cure from infection, which indicates that development of an effective vaccine against leishmaniasis is possible. Such protection may also be achieved by immunization with live attenuated parasites that do not cause disease. We have previously reported a protective response in mice, hamsters and dogs with *Leishmania donovani* centrin gene knock-out parasites (*LdCen*^*-/-*^), a live attenuated parasite with a cell division specific centrin1 gene deletion. In this study we have explored the effects of salivary protein LJM19 as an adjuvant and intradermal (ID) route of immunization on the efficacy of *LdCen*^*-/-*^ parasites as a vaccine against virulent *L*. *donovani*.

**Methodology/Principal Findings:**

To explore the potential of a combination of *LdCen*^-/-^ parasites and salivary protein LJM19 as vaccine antigens, *LdCen*^-/-^ ID immunization followed by ID challenge with virulent *L*. *donovani* were performed in hamsters in a 9-month follow up study. We determined parasite burden (serial dilution), antibody production (ELISA) and cytokine expression (qPCR) in these animals. Compared to controls, animals immunized with *LdCen*^*-/-*^ + LJM19 induced a strong antibody response, a reduction in spleen and liver parasite burden and a higher expression of pro-inflammatory cytokines after immunization and one month post-challenge. Additionally, a low parasite load in lymph nodes, spleen and liver, and a non-inflamed spleen was observed in immunized animals 9 months after the challenge infection.

**Conclusions:**

Our results demonstrate that an ID vaccination using *LdCen*^*-/-*^parasites in combination with sand fly salivary protein LJM19 has the capability to confer long lasting protection against visceral leishmaniasis that is comparable to intravenous or intracardial immunization.

## Author Summary

Leishmaniasis is a disease with a wide spectrum of clinical manifestations caused by different species of protozoa belonging to the *Leishmania* genus that are transmitted by sand fly vectors. Visceral infections of *Leishmania* cause significant mortality and morbidity and development of a vaccine to prevent leishmaniasis has become a high priority. We have previously reported that intravenous immunization with a live attenuated parasite vaccine comprised of *Leishmania donovani* parasites lacking the centrin gene conferred protection in mice, hamsters and dogs. In the current report, we describe the immunological response and associated protection to the ID immunization with attenuated parasites in combination with a sand fly salivary protein (LJM19). We observe that protection against experimental ID challenge with *L*. *donovani* resulting from ID immunization with live attenuated parasites in combination with LJM19 is comparable to intracardial immunization and offers improved protective immunity compared to immunization with salivary protein alone and non-immunized hamsters. This study supports the potential use of the genetically attenuated vaccine and a recombinant sand fly salivary protein for control of visceral leishmaniasis.

## Introduction

Leishmaniasis is a disease with a wide spectrum of clinical manifestations caused by different species of protozoa belonging to the *Leishmania* genus that are transmitted by sand fly vectors [1]. The disease causes high morbidity and significant mortality throughout the world, where 350 million people in 98 countries are at risk of contracting the infection. Moreover, approximately 1.0 to 1.5 million cases of cutaneous leishmaniasis (CL), and 200,000 to 500,000 cases of visceral leishmaniasis (VL), are registered annually [2].

VL is fatal if not treated [2]. The treatment of leishmaniasis is still based on the use of the parenteral administration of pentavalent antimonial compounds. However, side effects associated with the treatment and increased parasite resistance have made control and elimination of VL a serious challenge [3,4]. Therefore, the development of new strategies to prevent leishmaniasis has become a high priority [5].

The development of a vaccine for VL has been the focus of several research groups. Among the various types of vaccines, genetically modified live-attenuated vaccines provide the immunized host with diverse and complex antigens and induce a potent protective immunity in murine models [5,6]. Importantly live attenuated parasites cause no pathology in experimental infections [7–14], while inducing protection reflected by a significant reduction of parasite burden in animals challenged with virulent wild type strains [10,12,14–18].

We have previously reported on the *LdCen*^*-/-*^ parasites as a live attenuated candidate vaccine in several animal models [12,14,18]. Infection with *LdCen*^*-/-*^ was non-pathogenic i.e., safe and highly immunogenic in mice, hamsters and dogs [12,13,18]. In addition, immunization with *LdCen*^*-/-*^ induced protection against homologous challenge with wild type *L*. *donovani* and conferred cross-protection against infection with a heterologous challenge with *L*. *braziliensis*, *L*. *mexicana* and *L*. *infantum* [14,18]. However, previous studies with *LdCen*^*-/-*^ parasites as immunogens were performed without any adjuvants. Since the adjuvants can activate a range of innate immune pathways it is difficult to predict on an empirical basis which adjuvant will work most effectively with live attenuated parasites. Since the adaptive response is the primary determinant of protective immunity generated by vaccination, immunomodulatory reagents that could supplement *LdCen*^*-/-*^ induced immunity without causing rapid elimination of the vaccine antigen due to innate immune reactions could make *LdCen*^*-/-*^ more effective as an anti-*Leishmania* vaccine.

Saliva from sand flies contains potent pharmacologic components that facilitate blood meal acquisition and modulates the host inflammatory and immune responses [19,20]. Arthropod vector saliva also plays an important role in pathogen transmission from the sand fly to the vertebrate host [21]. Recent reports have shown the importance of some salivary proteins from sand fly vectors such as LJM19, LJM11 or LJM17 as potential targets for vaccine development against *Leishmania* infection [11,19,22–30]. A specific immune response against salivary proteins has been reported in various animal models. For example, hamsters immunized with plasmid DNA coding for LJM19, a *Lu*. *longipalpis* salivary protein, protected them from disease after challenge with wild type *Leishmania infantum chagasi* parasites plus saliva through the induction of a LJM19-specific immune response [26]. By comparison, salivary protein LJM11 provided partial protection that was not long lasting against virulent challenge [26]. Importantly, immunization with LJM19 induced higher ratios of IFN-γ/IL-10 and IFN- γ/TGF-β in the spleen, conditions consistent with a Th1 polarization [26,28]. These results suggested that salivary gland proteins such as LJM19 could be a potent supplement to the protective immunity with live attenuated *Leishmania* parasites.

In our previous studies, we have tested different routes of immunization, including intravenous (tail vein), intracardial and subcutaneous [12,13]. However, intradermal immunization can offer improved protective immunity and simplify the logistics of delivery as was previously demonstrated [26,28,29,31,32]. Therefore, it is of value to evaluate live attenuated parasite vaccines for their efficacy following intradermal immunization.

Since our previous studies have shown that exposure to live attenuated parasites injected by intravenous or intracardial routes and without an adjuvant induced a strong protective immunity, we asked whether an intradermal immunization with *LdCen*^*-/-*^ parasites in combination with LJM19 could further enhance vaccine induced protection. In the present study, we report for the first time the immunogenicity and protection outcome in hamsters intradermally primed with salivary protein LJM19 and boosted with genetically modified live attenuated *L*. *donovani* parasites (*LdCen*^*-/-*^) in combination with recombinant LJM19. Immunized hamsters demonstrated a strong immune response comparable to that of intracardial immunization with *LdCen*^*-/-*^ and resulted in long term protection against infection with virulent *L*. *donovani* parasites.

## Materials and Methods

### Animals

Two-month-old female Syrian golden hamsters (*Mesocricetus auratus*) were obtained from the Harlan Laboratories and kept in the Food and Drug Administration (FDA) animal facility. The experimental procedures used in this study were reviewed and approved by the Animal Care and Use Committees of the FDA and the National Institute of Allergy and Infectious Diseases (NIAID).

### Ethics statement

The animal protocol for this study has been approved by the Institutional Animal Care and Use Committee at the Center for Biologics Evaluation and Research, US FDA (ASP 1995#26). Further, the animal protocol is in full accordance with ‘The guide for the care and use of animals’ as described in the US Public Health Service policy on Humane Care and Use of Laboratory Animals 2015 (http://grants.nih.gov/grants/olaw/references/phspolicylabanimals.pdf).

### Sand flies and preparation of SGH

*Lu*. *longipalpis* sand flies, Jacobina strain were reared at the Laboratory of Malaria and Vector Research, NIAID. Salivary glands were dissected from 5- to 7-day-old females and stored in PBS at -70°C. Before use, salivary glands were sonicated and centrifuged at 12,000×*g* for 2 min. The supernatant was collected and used immediately.

### Intradermal immunization (*LdCen*^*-/-*^ ± LJM19) and challenge (parasites and SGH)

*LdCen*^*-/-*^ and *L*. *donovani* (Ld1S) promastigotes were grown at 26°C in medium 199 supplemented with 20% FCS. Three- to 4-month-old hamsters were immunized intradermally in the ear at two-week intervals between immunizations, using a 29-gauge needle (BD Ultra-Fine) in a volume of 20μl, using the following protocols. Group 1: prime with 2μg of LJM19 protein and boost with 10^7^ stationary phase *LdCen*^*-/-*^promastigotes plus 2μg of LJM19. Group 2: 10^7^ stationary phase *LdCen*^*-/-*^ promastigotes via intracardial injection. Group 3: 2μg of LJM19 protein (two times). Group 4: BSA (control) (S1 Fig). Each experimental group consisted of 6 hamsters. Five weeks after the last immunization, the animals were challenged ID with 10^5^ stationary phase Ld1S promastigotes in combination with 0.5 pairs SGH.

### Determination of parasite burden

At 5 weeks post immunization, and 1 and 9 months post challenge, the parasite load was measured in the ear, lymph node (from challenged ear), spleen and liver by the limiting dilution assay as previously described [33].

### Antibody detection

Whole blood from immunized hamsters was collected at the indicated time points before sacrifice in 1.1mL microcentrifuge tubes with serum gel-clotting activator (Sartesdt, GE). The serum was separated by centrifugation and used in IgG assays. Total IgG, IgG_1_ and IgG_2_ responses to *L*. *donovani* soluble antigens were measured by ELISA as described [34]. The following clones were used in the study. IgG cocktail (Catalog# 554010); IgG1 (clone-G94-56); IgG2 (clone-G192-3) (BD Biosciences).The cut-off value of reactivity for SLA antigens was calculated as the mean plus 2 SD of the OD values observed in naive controls. Sera from 9 naïve hamsters were used for determining cut-off values.

### Cytokine determination by real-time PCR

Splenocytes were collected from hamsters, macerated, lysed in Trizol for RNA extraction. Total RNA was extracted from the ear, lymph node (superficial parotid lymph node), spleen and liver of infected hamsters using TRIzol reagent (Invitrogen). First-strand cDNA synthesis was performed with ≈1–2μg of RNA using a Transcriptor High Fidelity cDNA Synthesis Kit (Roche). Amplification conditions consisted of an initial pre-incubation at 95°C for 10 min, followed by amplification of the target DNA for 40 cycles of 95°C for 15 s and 60°C for 1 min with the LightCycler 480 (Bio-Rad). The efficiency of each reaction was determined. The expression levels of genes of interest were normalized to β-Actin levels. The results are expressed in fold change of 2^-ΔCt^ over control.

### Oligonucleotide primers and probes

Oligonucleotide primers used for real time PCR were: β Actin, reverse, ACA GAG AGA AGA TGA CGC AGA TAA TG, forward, GCC TGA ATG GCC ACG TAC A; IFN-γ, reverse, TGT TGC TCT GCC TCA CTC AGG, forward, AAG ACG AGG TCC CCT CCA TTC; TNF-α, reverse, TGA GCC ATC GTG CCA ATG, forward AGC CCG TCT GCT GGT ATC AC; IL-10, reverse, GGT TGC CAA ACC TTA TCA GAA ATG, forward, TTC ACC TGT TCC ACA GCC TTG; IL-4, reverse, ACA GAA AAA GGG ACA CCA TGC A, forward, GAA GCC CTG CAG ATG AGG TCT; IL-12/IL-23p40, reverse, AAT GCG AGG CAG CAA ATT ACT C forward, CTG CTC TTG ACG TTG AAC TTC AAG, iNOS, reverse, ACC ACA CAG CCT CCG AGT CC, forward, CTG CCA GAT GTG GGT CTT CC. The primers and probes were synthesized at the Center for Biologics and Evaluation and Research, FDA Core facility.

### Statistical analysis

Statistical analysis was performed using GraphPad Prism 5.0 software (GraphPad Software Inc., USA). Non-parametric Kruskal-Wallis test followed by Dunns test were used to compare data from four groups (G1, G2, G3 and G4). Differences were considered significant when a *p* value ≤ 0.05 was obtained.

## Results

### Persistence of *LdCen*^*-/-*^ in intradermally immunized animals

**Fig 1:**
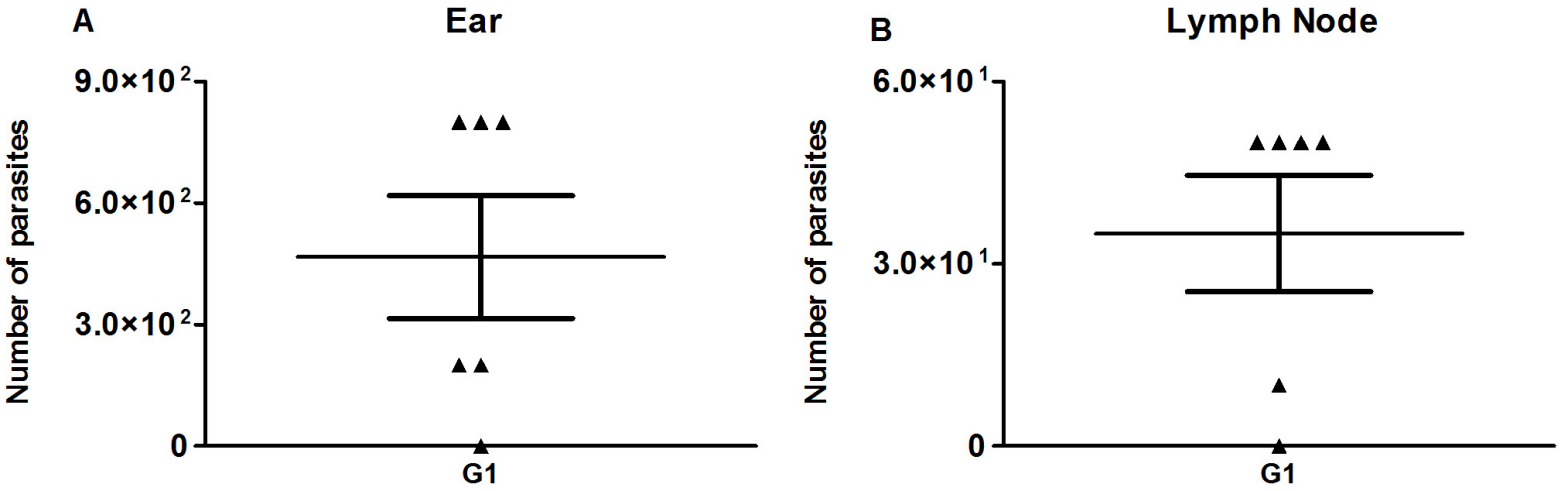
Parasite burden 5 weeks after immunization. Detection of parasites by limiting dilution assays and expressed as number of parasites per organ after 5 weeks of immunization with *LdCen*^*-/-*^ in the ear (A) and lymph node (B) of group 1 (G1).

Since the efficacy of genetically modified *Leishmania* parasites in general, and *LdCen*^*-/-*^ parasites in particular have not been tested through an intradermal route, we sought to determine the persistence of *LdCen*^*-/-*^ parasites at the site of injection and its dissemination to other organs where relevant immune reactions might occur. Thus, we measured the parasite load in the ear, lymph node, spleen and liver 5 weeks post immunization (5wpi) in hamsters primed with LJM19 and boosted with LJM19 plus *LdCen*^*-/-*^ (G1). We observed an average of 300 and 30 viable *LdCen*^*-/-*^ parasites in the ear and draining lymph node, respectively, measured by limiting dilution (Fig 1A and 1B). However, we could not recover any *LdCen*^*-/-*^ parasite from the spleen and liver from either G1 or from animals immunized with *LdCen*^*-/-*^ intracardially.

### Prime/boost intradermal immunization with LJM19 and *LdCen*^*-/-*^ plus LJM19 induced a strong anti-*Leishmania* antibody response

**Fig 2:**
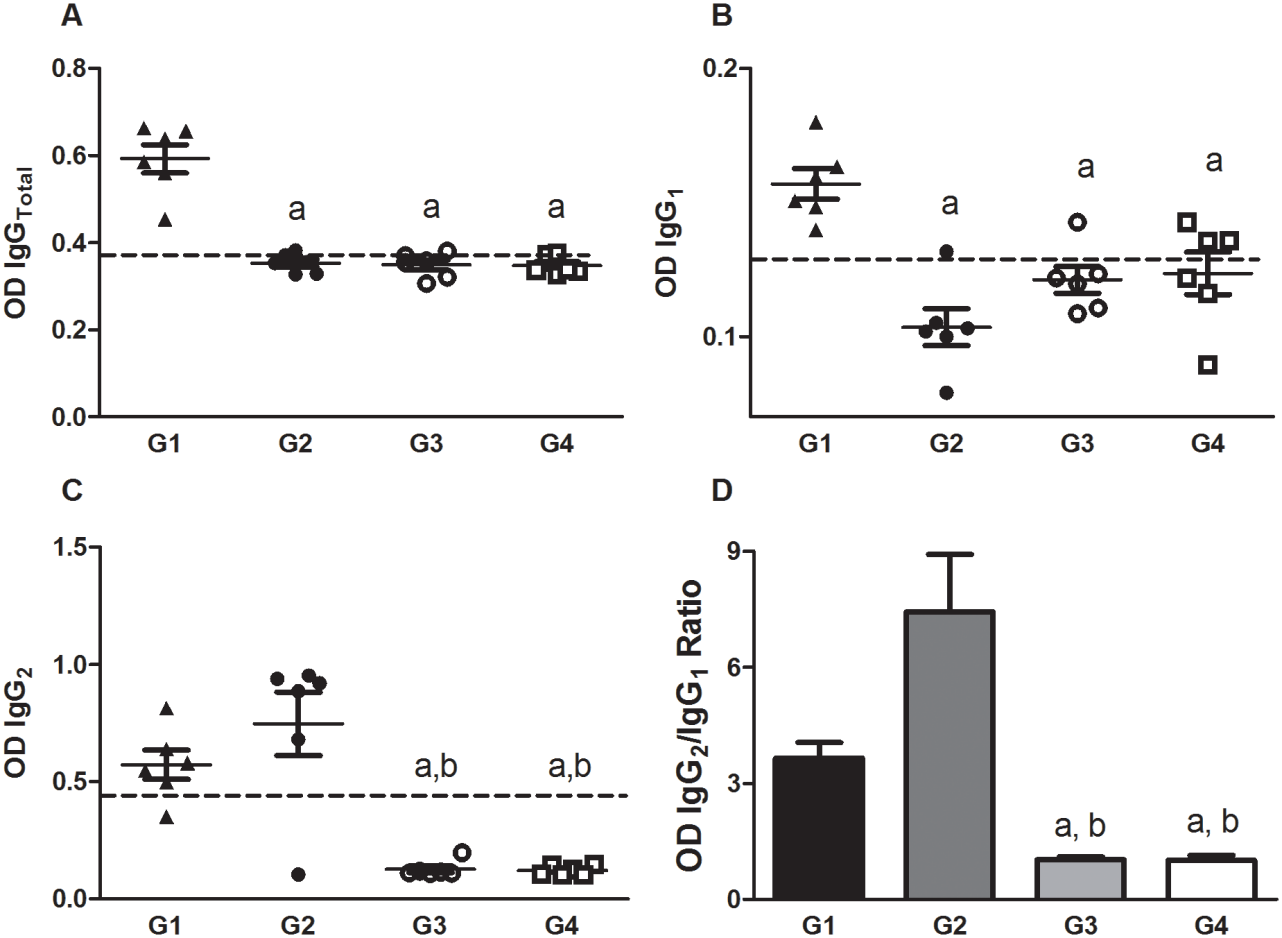
Specific antibody production 5 weeks after immunization. ELISAs using plates coated with SLA from *L*. *donovani* were performed to detect production of total IgG (A), IgG_1_ (B), IgG_2_ (C) and the ratio IgG_2_/IgG_1_ (D). The OD values representing antibody concentration are shown on the *y*-axis, and the error bars indicate the standard deviation. Dotted lines represent the cut-off value. Statistical differences (*p*<0.05) are indicated in letters (a: G1 and b: G2).

The analysis of sera from hamsters primed with LJM19 and boosted with *LdCen*^*-/-*^ plus LJM19 indicated the occurrence of strong *Leishmania*-specific antibody responses. The SLA-specific IgG_Total_ and IgG_1_ production was significantly higher for immunized animals in G1 when compared to animals immunized IC with *LdCen*^*-/-*^ (IC, G2), at 5wpi (*p*<0.05) (Fig 2A and 2B). However, increased levels of IgG_2_ were detected in G1 and G2 groups compared to G3 and control group G4 (*p*<0.01 and *p*<0.001, respectively; Fig 2C) that show base line reactivity. Importantly, the IgG_2_/IgG_1_ ratio was significantly higher in G1 and G2 groups compared to G4, the control group (*p*<0.05) and to G3 (*p*<0.05) that received LJM19 alone (Fig 2D). Of note, the IgG_2_/IgG_1_ ratio was higher in G2 compared to G1 (*p*<0.05) (Fig 2D).

### Local expression of cytokines in the ear of immunized hamsters

**Fig 3:**
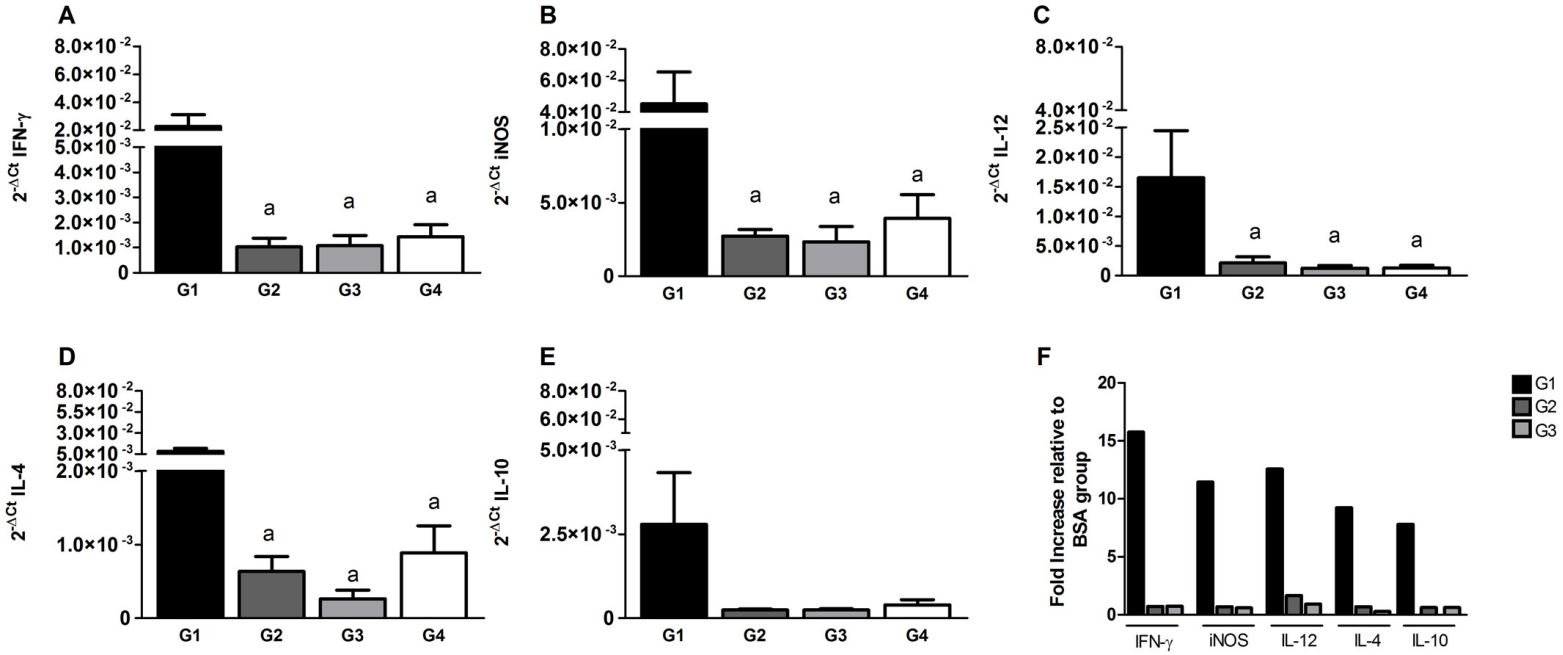
Cytokine responses 5 weeks after immunization with *LdCen*^*-/-*^ parasites and LJM19. Immune response in the ear tissue of immunized hamsters was measured. The expression of mRNA encoding IFN-γ (A), iNOS (B), IL-12 (C), IL-4 (D) and IL-10 (E) was evaluated by qPCR. The data were normalized to β-Actin expression. (F) Fold increase of the cytokines compared to BSA (G4) group. Statistical differences (*p*<0.05) are indicated in letters (a: G1).

In order to evaluate the protective immunity induced after ID immunization, we analyzed the mRNA expression of both Th1 and Th2 cytokines (IFN-γ, iNOS, IL-12/IL-23p40, IL-4 and IL-10) in the ear, the site of injection at 5wpi. The IFN-γ, iNOS and IL-12 expression levels were significantly higher in immunized hamsters in G1 compared with G2, G3 and G4 groups (*p*<0.05; Fig 3A,3B and 3C, respectively). Expression of IL-4 and IL-10 cytokines was also higher in G1 animals after immunization, when compared to animals in groups G2, G3 and G4 (*p*<0.05; Fig 3D and 3E, respectively). However, the level of IFN-γ was higher than either IL-10 or IL-4 in G1 animals. Additionally, G1 animals showed up to a ~16-fold, ~12-fold and ~13-fold increase in IFN-γ, iNOS and IL-12 respectively, compared to BSA-immunized control group G4 (Fig 3F). Similarly, mRNA levels of IL-4 and IL-10 were up-regulated ~9- and 8-folds, respectively, in G1 compared to G4 animals.

### Intradermal immunization with *LdCen*^*-/-*^ parasites results in control of the parasite load of virulent *L*. *donovani* early in infection

**Fig 4:**
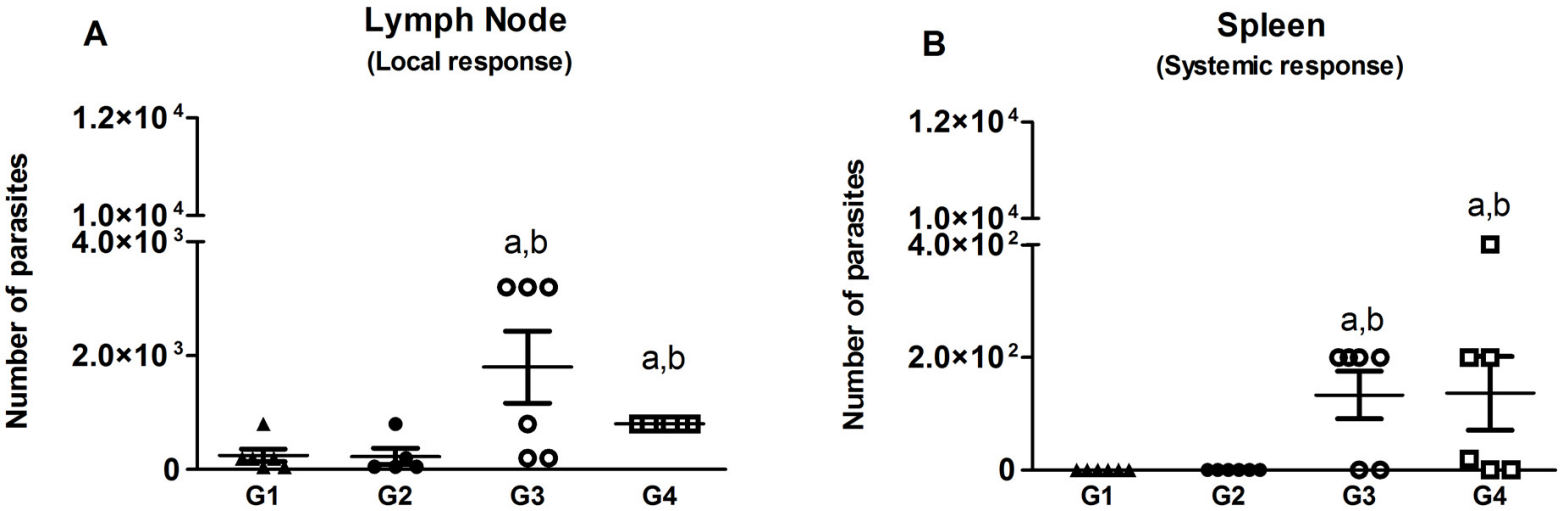
Parasite burden 1 month after challenge. Hamsters were challenged in the left ear with virulent *L*. *donovani* and presence of parasites was detected 1 month after challenge. The draining lymph node (A) and spleen (B) were used in limiting dilution assay and data expressed as number of parasites/organ. Statistical differences (*p*<0.05) are indicated in letters (a: G1 and b: G2).

The number of viable *L*. *donovani* parasites was determined by the limiting dilution assay in the draining lymph node and spleen of immunized hamsters a month post challenge (mpc) (Fig 4). The number of live parasites in the lymph node (Fig 4A) and in the spleen (Fig 4B) were significantly lower (*p*<0.05) in the G1 and G2 groups either immunized with LJM19 then boosted with *LdCen*^*-/-*^ plus LJM19 or immunized with *LdCen*^*-/-*^ alone respectively, when compared with the groups of hamsters that received LJM19 alone or BSA alone.

### *Leishmania*-specific IgG isotypes in immunized hamsters post challenge

**Fig 5:**
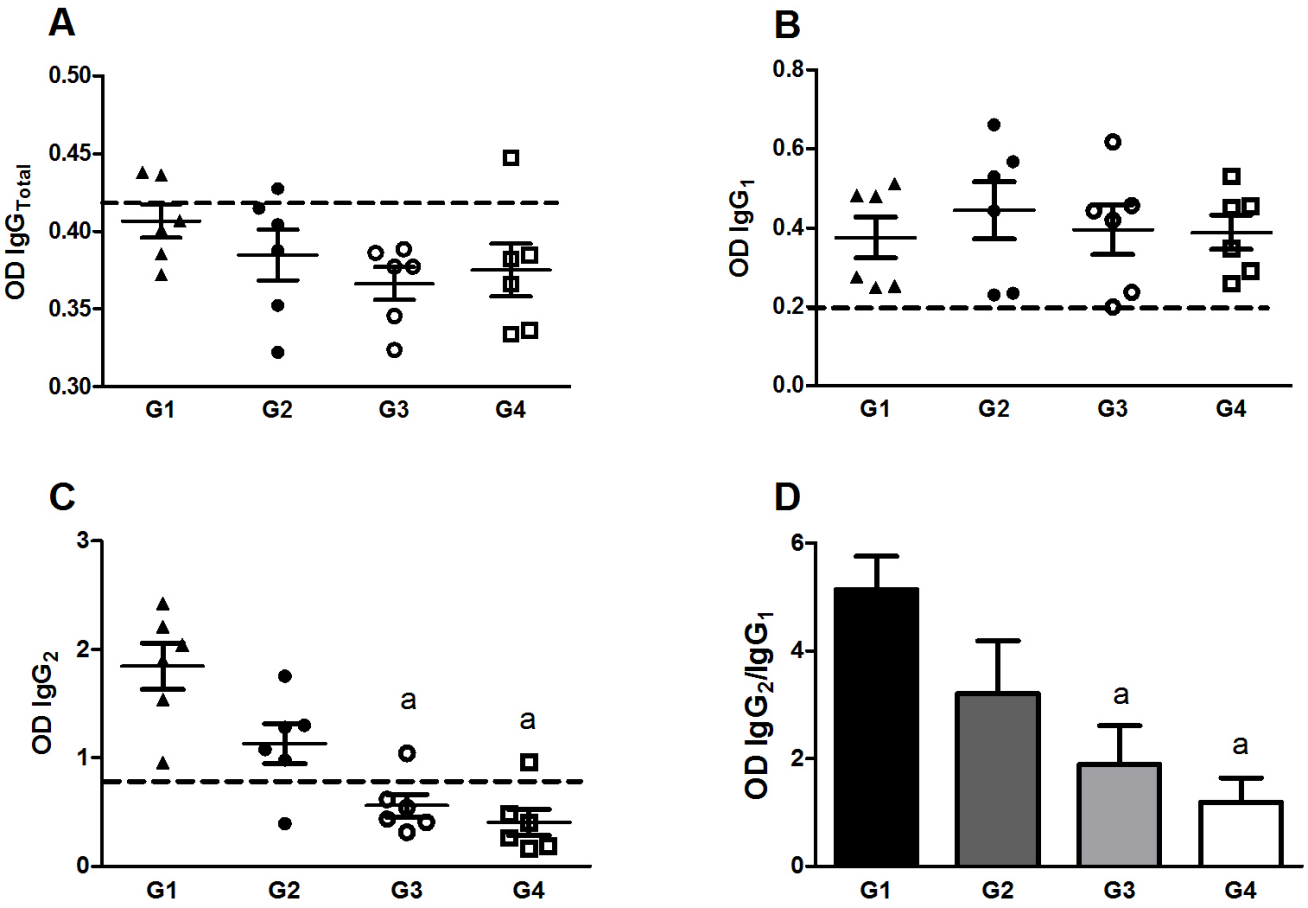
Specific antibody production 1 month after challenge. ELISAs using plates coated with SLA from *L*. *donovani* were performed to detect production of total IgG (A), IgG_1_ (B), IgG_2_ (C) and the ratio IgG_2_/IgG_1_ (D). The antibody OD values are shown on the *y*-axis, and the error bars indicate the standard deviation. Dotted lines represent the cut-off value. Statistical differences (*p*<0.05) are indicated in letters (a: G1).

We measured antibody levels in the sera of immunized animals one mpc. No difference was observed in the levels of IgG_Total_ and IgG_1_ between the groups (Fig 5A and 5B). The level of IgG_2_ was elevated in G1 immunized animals when compared to G3 and G4 hamsters (*p*<0.05 and *p*<0.01, respectively; Fig 5C). In addition, G1 group presented a significantly higher IgG_2_/IgG_1_ ratio in comparison to G3 and G4 control groups (*p*<0.05 and *p*<0.01, respectively) (Fig 5D), indicative of a Th1-type immune response post challenge.

### The early immune response in lymph nodes, spleen and liver of immunized animals post challenge

**Fig 6:**
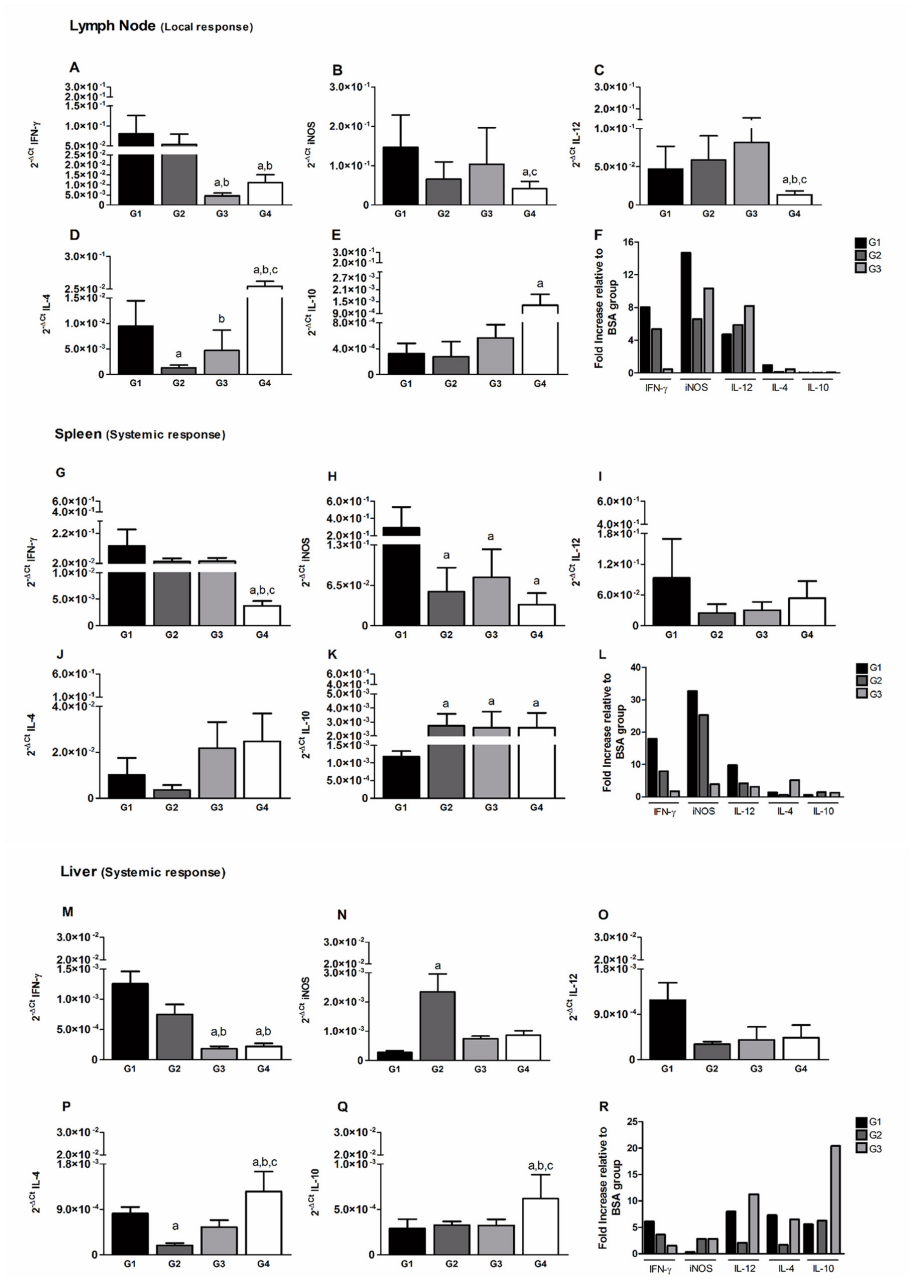
Cytokines response 1 month after challenge. Immune response in the lymph nodes of hamsters immunized with *LdCen*^*-/-*^ and LJM19 by qPCR (A to E). The mRNA expression of IFN-γ (A), iNOS (B), IL-12(C), IL-4 (D), and IL-10 (E) was evaluated. The data were normalized to β-actin expression. Fold increase of the cytokines compared to BSA (G4) group (F). Immune response in the spleens of hamsters immunized with *LdCen*^*-/-*^ and LJM19 by qPCR (G to K). The mRNA expression of IFN-γ (G), iNOS (H), IL-12(I), IL-4 (J), and IL-10 (K) was evaluated. The data were normalized to β-actin expression. Fold increase of the cytokines compared to BSA (G4) group (L). Immune response in the livers of hamsters immunized with *LdCen*^*-/-*^ and LJM19 by qPCR (M to Q). The mRNA expression of IFN-γ (M), iNOS (N), IL-12(O), IL-4 (P), and IL-10 (Q) was evaluated. The data were normalized to β-actin expression. Fold increase of the cytokines compared to BSA (G4) group (R). Statistical differences (p<0.05) are indicated in letters (a: G1, b: G2 and c: G3).

The mRNA expression level of Th1 and Th2 cytokines was estimated by qRT-PCR one mpc. In the lymph node (LN), G1 and G2 groups presented a high expression of IFN-γ, when compared to G3 and G4 groups (*p*<0.01) (Fig 6A). A moderate increase in the expression levels of iNOS mRNA transcripts in LN was observed only in G1, and G3 immunized hamsters compared to G4 group (*p*<0.05) (Fig 6B). Interestingly, cytokine IL-12 mRNA levels in LN were significantly higher in G1, G2 and G3 groups compared to the G4 control group (*p*<0.001) (Fig 6C). Concomitantly, the mRNA levels of the Th2 cytokines IL-4 and IL-10, primarily regulatory cytokine, were higher in LN of G4 control animals in comparison to G1, G2 and G3 immunized groups (*p*<0.05) (Fig 6D and 6E). Additionally, IFN-γ, iNOS and IL-12 from G1 were significantly up-regulated by ~8 folds, ~14 folds and ~5 folds, respectively, compared to G4 group (Fig 6F).

In the spleen, IFN-γ was up-regulated one mpc in groups G1 (*p*<0.001), G2 (*p*<0.01) and G3 (*p*<0.01), compared to control group (G4) (Fig 6G) In addition, G1 presented a higher expression of iNOS when compared to G2, G3 and G4 after challenge (*p*<0.001, *p*<0.005 and *p*<0.01, respectively) (Fig 6H). Interestingly, IL-12 mRNA levels were not significantly different in spleens from animals of the 4 groups though G1 animals exhibited a trend for increased IL-12 expression (Fig 6I). The IL-4 expression was decreased in G1 and G2 groups, but not significantly (Fig 6J) compared to G3 and G4. However, there was a significant reduction in IL-10 expression (*p*<0.05) in G1 when compared to G2, G3 and G4 groups (Fig 6K). The IFN-γ, iNOS and IL-12 mRNAs from G1 were significantly up-regulated by ~19 folds, ~32 folds and ~10 folds, respectively, compared to G4 group (Fig 6L).

In the liver, IFN-γ expression was upregulated one mpc in G1 and G2 groups in comparison with G3 and G4 animals (*p*<0.01) (Fig 6M). However, iNOS expression was significantly higher in G2 compared to G1, G3 and G4 (Fig 6N). Liver cells from group G1 immunized hamsters induced a significantly high expression of IL-12 (Fig 6O). IL-4 expression was significantly higher in G4 animals compared to G1, G2 and G3 hamsters (*p*<0.01), and lower in G2 group compared to G1 group, but was not significantly different compared to G3 group (Fig 6P). On the other hand, hamsters from control group G4 showed a significant up-regulation (*p*<0.001) in IL-10 expression compared to G1, G2 and G3 (Fig 6Q). The IFN-γ, iNOS and IL-12 mRNAs from G1 were moderately up-regulated by ~1.3 folds, ~0.39 folds and ~1.2 folds, respectively, compared to control group G4 (Fig 6R).

### Prime/boost immunization with LJM19 and *LdCen*^-/-^ plus LJM19 offered long term protection against a virulent *L*. *donovani* challenge in hamsters

**Fig 7:**
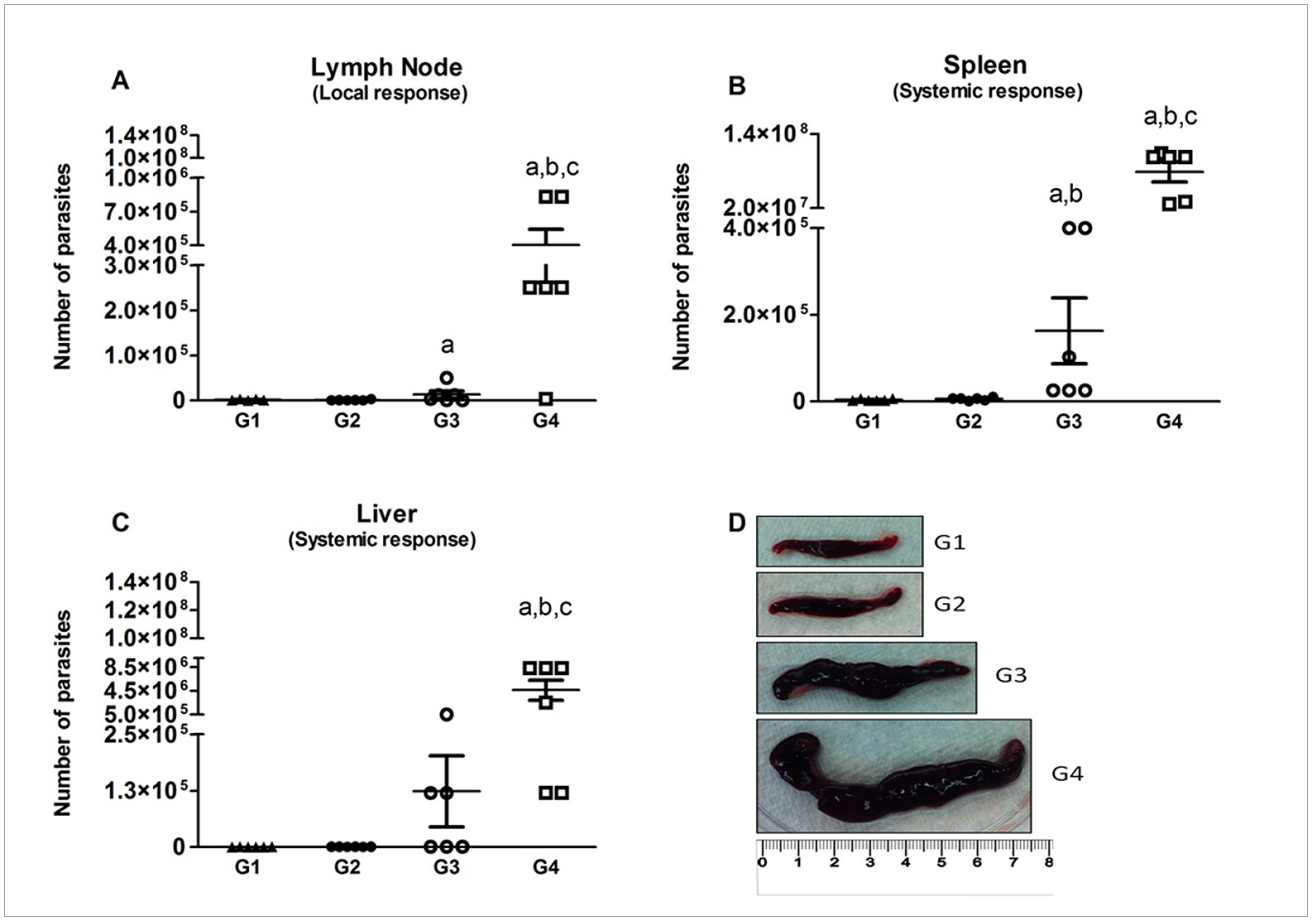
Parasite burden 9 months after challenge. Hamsters were challenged in the left ear with virulent *L*. *donovani* and presence of parasites was detected 9 months after challenge. The draining lymph node (A), spleen (B) and liver (C) were used in limiting dilution assay and data expressed as number of parasites/organ. Statistical differences (p<0.05) are indicated in letters (a: G1, b: G2 and c: G3). (D) Comparison of spleen size 9 months after challenge. The median of spleen size of hamsters of G1, G2, G3 and G4 was measured in centimeter. A representative sample from 6 immunized and challenged hamsters is shown.

The animals in G1 and G2 groups showed robust protection 9 mpc as evident by a significant decrease in the lymph node parasite load (Fig 7A), spleen (Fig 7B) and liver (Fig 7C) in comparison to G4 animals (*p*<0.0001). Immunization with salivary gland protein LJM19 alone (G3) also provided significant protection (*p*<0.01), albeit weaker than that observed in animals in groups G1 and G2, compared to G4 BSA immunized animals.

Further, we wanted to test whether *LdCen*^*-/-*^ immunization causes re-establishment of homeostatic conditions by comparing the spleen sizes. Both G1 and G2 presented a non-inflamed spleen (median 3.4 and 3.5cm, respectively), as compared to a highly inflamed spleen in the control group G4 (median 7.9cm) (Fig 7D). G3 animals showed an intermediate spleen size (median 5.2 cm).

## Discussion

Previous work from our laboratories has shown that the *LdCen*^*-/-*^ live attenuated vaccine is immunogenic in mice, hamsters and dogs [12,13,18]. Similarly, LJM19 protein from saliva of the vector *Lu*. *longipalpis* protected hamsters against challenge with *L*. *infantum* and *L*. *braziliensis* [26,28]. In the present work, we examined the value of combining the two immunization strategies for their potential to elicit protective immune responses in a hamster model of *Leishmania donovani* infection. ID needle inoculation of the ear has been extensively employed as the route of infection that most closely replicates the physiological ID and intra-epidermal deposition of parasites by the bite of an infected sand fly [33,35–37]. Additionally, the ID route presents the most practical route for vaccine delivery [26,28,29,31,32]. We hypothesized that a prime/boost strategy with LJM19 followed by *LdCen*^*-/-*^ parasites plus LJM19, all delivered intradermally, would induce long-lasting protective immunity against *L*. *donovani* particularly since *LdCen*^*-/-*^ parasites can undergo limited replication in the immunized host and provide an array of antigens very similar to those produced by a virulent parasite. As such, in this prime/boost protocol, priming with LJM19 would generate a specific adaptive immune response to the sand fly salivary gland protein as was observed in previous studies [26,28] that could result in a potent supplement to the specific adaptive immune response to antigens of the *LdCen*^*-/-*^ parasites.

We had previously observed *LdCen*^*-/-*^ parasites in the spleen up to 5 wpi after intracardial (IC) injection [12]. In the current study, we observed parasites in the immunized ear and the draining lymph node but not in the spleen, at 5wpi after ID injection, suggesting that either the parasites take a longer time to disseminate to the viscera and reach the spleen or alternatively they do not visceralize. Of interest, recent studies with dermotropic parasite strains (*L*. *donovani* isolated from a cutaneous lesion and *L*. *major*) that fail to persistently visceralize nevertheless produced protective immunity in a low-dose infection followed by challenge with *L*. *donovani* and *L*. *infantum* in mouse models [38,39]. This suggests that visceralization may not be a necessary pre-condition for protective immunity against VL to develop in the immunized mice. Consistent with this hypothesis, our results indicate that since the draining lymph nodes represent the immunological niche where relevant reactions between APCs that acquired the antigens and naïve T cells could occur, recovery of attenuated parasites from the lymph nodes 5 weeks post immunization suggested that parasite persistence, i.e. antigen availability, was adequate for protective immunity to be established.

Immunization of hamsters with attenuated parasites associated with LJM19 protein elicited a biased Th1-type immune response at 5wpi at the site of injection. As expected, immunization by LJM19 alone provided protection against *L*. *donovani* parasites, however, it was considerably weaker compared to the one observed following a prime/boost ID immunization with LJM19 followed by LJM19 and *LdCen*^*-/-*^ or an IC immunization with *LdCen*^*-/-*^. Of note, boosting with *LdCen*^*-/-*^ along with LJM19 protein through the ID route resulted in a higher pro-inflammatory response compared to immunization with *LdCen*^*-/-*^alone through the IC route. Indeed, immunization with *LdCen*^*-/-*^ through the intravenous (IV) route in mice and IC route in hamsters [12] and subcutaneous route in dogs [13] has been shown to promote a pro-inflammatory response, with the presence of IL-12p40, IFN-γ, iNOS and TNF-α. Additionally, our finding that increased levels of IL-4, IFN-α, iNOS and IL-12/IL-23p40 in immunized hamsters would suggest a mixed immune response (Th1 biased) triggered by LdCen^-/-^ vaccination, as we observed in dogs immunized previously [18,38,40]. Our results suggest that the ID mode of immunization, at least in combination with LJM19, is equally efficacious compared to the IC mode. Additionally, the high ratio of IgG_2_/IgG_1_ observed in groups G1 and G2 is considered an additional immune biomarker of protection [5,41–43].

During natural transmission, an infected sand fly deposits saliva and parasites into the skin of the host while feeding. To mimic the natural mode of infection with *Leishmania*, we injected *L*. *donovani* wild type parasites into the ear of hamsters along with sand fly salivary gland extract. After one month of infection, hamsters immunized with *LdCen*^*-/-*^ either alone (IC) or with LJM19 protein (ID) demonstrated a reduced parasite burden in the lymph node and spleen. In our study, similar to the response observed post-immunization, challenged hamsters presented a significant increase of IgG_2_ production, and a high ratio of IgG_2_/IgG_1_, as well as an enhanced production of IFN-γ and iNOS. Higher levels of IgG_2_ might also contribute to pathogen clearance in vaccinated animals [44,45].

Previously, it was reported that iNOS and concomitant high levels of NO were produced by macrophages in protected mice vaccinated with attenuated parasites after challenge with *L*. *donovani* [12,14]. In the present study hamsters immunized with either *LdCen*^*-/-*^ alone or in association with recombinant LJM19 displayed increased IFN-γ and iNOS expression in lymph nodes and spleen one month after challenge with wild type parasites. Of importance, five weeks post-immunization we observed a higher production of IL-12 only in animals immunized with *LdCen*^*-/-*^ in association with recombinant LJM19. It can be speculated that LJM19 might be pre-conditioning the innate immune arm and thus allowing the antigen presenting cells such as DCs to produce IL-12 that is necessary for initiating a strong adaptive Th1 cell immunity. Certainly, the ability of LJM19 to produce a Th1 response in hamsters has been previously demonstrated [26]. As such, it may be argued that LJM19 might be enhancing the immunogenicity of *LdCen*^*-/-*^ as a vaccine. An increased IFN-γ/IL-10 ratio has been observed when DNA vectors expressing KMP11 along with LJM19 were used as immunogens compared to either KMP11 or LJM19 alone at 5 months post challenge [46]. This increased IFN-γ/IL-10 ratio did not result in reduced splenic parasite burden between KMP11+LJM19 and either antigen or LJM19 alone groups at 5 months post challenge. Further in their study the authors also observed increased IFN-γ/IL-10 ratio after 5 months of infection in non-immunized animals which does not explain the role of increased IFN-γ levels in protection. The observed differences between da Silva [46] and our study in splenic parasite burden could be due to the ability of the live attenuated parasites to induce sustained immunological reactions (Fig 6G and 6K) because of a longer availability of a multitude of antigens compared to recombinant antigens that tend to have limited availability and diversity that is reflected in parasite control up to 9 months post challenge. Further, in the *LdCen*^*-/-*^ immunized animals (G1 and G2) down-regulation of IL-10 and a concomitant increase in IL-12 in lymph node, spleen and liver may explain the greater parasite killing observed at the challenge site. In murine and human VL, production of Th1 cytokines is desirable for resolution of infection [47–50]. In addition, IL-12 results in the generation of Th1 cells that produce both IFN-γ and IL-12, thus favoring the development of a protective cellular immune response against *Leishmania* [51–53]. An important consideration is that the sustained Th1-type immune response and long-term protection generated here against *L*. *donovani* after ID injection of *LdCen*^*-/-*^ parasites in combination with LJM19 is comparable to that observed following IC or intravenous immunization with *LdCen*^*-/-*^ parasites alone [12,14]. A similar induction of Th1 immunity was also observed in dogs immunized subcutaneously with *LdCen*^*-/-*^ parasites and challenged with *L*. *infantum* [18]. At 9 months post challenge, the parasite load in the lymph nodes and in the spleen was significantly reduced in all the immunized groups compared to the control group. The control of parasitemia in the spleen translated into lack of splenomegaly in immunized and challenged animals compared to control challenged animals. Importantly, the significant reduction of parasitemia after immunization with LJM19 alone in our current study argues that LJM19 contributes to the observed protection in G1 hamsters. This is corroborated by Gomes at al. [26] who observed a decrease in parasite load in the spleen and liver in hamsters immunized with LJM19 after 2 and 5 months post I.D. inoculation of *L*. *infantum chagasi* with sand fly salivary gland homogenate. Additionally, in an independent study, Tavares et.al [28] showed that hamsters immunized with LJM19 induced protection against infection with *L*. *braziliensis*. Taken together our data indicate the induction of a long-lasting protective immune response in the spleen, liver and lymph nodes in hamsters immunized intradermally with LJM19 and *LdCen*^*-/-*^ after challenge with virulent parasites and reveal that a stronger immune response is elicited when *Leishmania donovani* live attenuated parasites are combined with a salivary gland protein. In summary, we have demonstrated the capability of a combined vaccine composed of live attenuated *LdCen*^*-/-*^ parasite and a defined salivary gland protein from *Lu*. *Longipalpis* (LJM19) delivered intradermally to confer strong long-lasting protection against *L*. *donovani* infection in a hamster model.

## Supporting Information

S1 Fig Scheme of immunization and challenge protocol. (TIF)
