# Supplementary figures and images for: Intradermal Immunization of Leishmania donovani Centrin Knock-Out Parasites in Combination with Salivary Protein LJM19 from Sand Fly Vector Induces a Durable Protective Immune Response in Hamsters
Source: PLoS Negl Trop Dis. 2016 Jan 11;10(1):e0004322. doi: 10.1371/journal.pntd.0004322 (PMC4708988; doi:10.1371/journal.pntd.0004322)

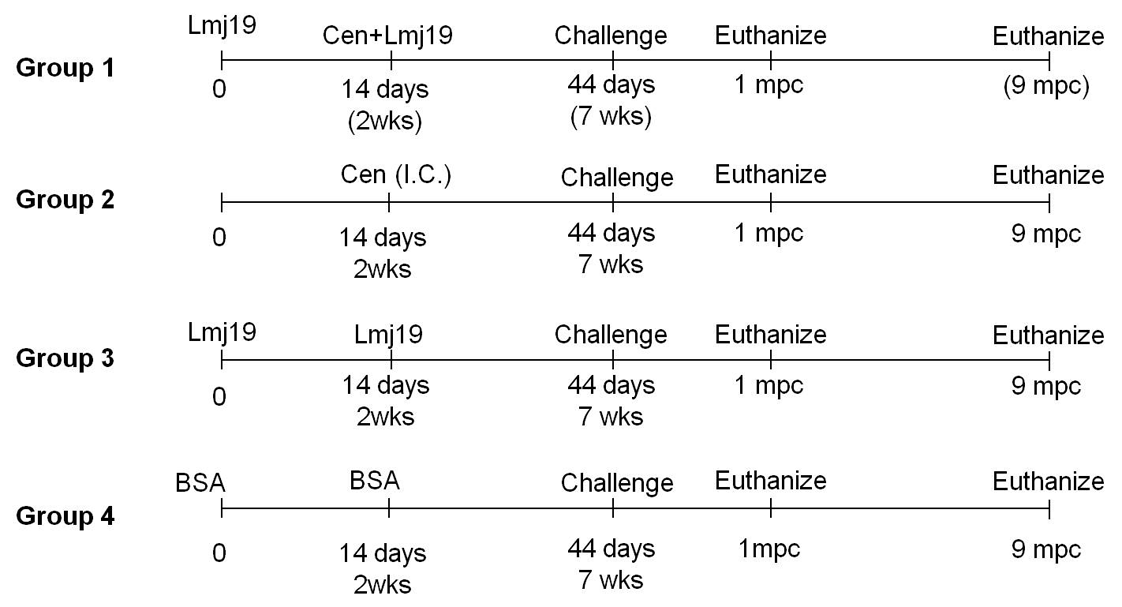

Supplement: S1 Fig — (TIF) [file pntd.0004322.s001.tif]
